# Dietary grape polyphenol resveratrol increases mammary tumor growth and metastasis in immunocompromised mice

**DOI:** 10.1186/1472-6882-13-6

**Published:** 2013-01-08

**Authors:** Linette Castillo-Pichardo, Luis A Cubano, Suranganie Dharmawardhane

**Affiliations:** 1 Department of Biochemistry, School of Medicine, University of Puerto Rico, Medical Sciences Campus, PO Box 365067, San Juan, Puerto Rico; 2 Department of Pathology and Laboratory Medicine, Universidad Central del Caribe School of Medicine, Bayamon, Puerto Rico; 3 Department of Anatomy and Cell Biology, Universidad Central del Caribe School of Medicine, Bayamon, Puerto Rico

**Keywords:** Breast cancer, Resveratrol, Metastasis, Grape polyphenols, Rac

## Abstract

**Background:**

Resveratrol, a polyphenol from grapes and red wine has many health beneficial effects, including protection against cardiovascular and neurodegenerative diseases and cancer. However, our group and others have provided evidence for a dual cancer promoting or inhibitory role for resveratrol in breast cancer, dependent on estrogenic or antiestrogenic activities. Moreover, much of the inhibitory effects of resveratrol have been reported from studies with high non-physiological concentrations.

**Methods:**

We investigated the effects of a range of concentrations (0.5, 5, 50 mg/kg body weight) of resveratrol on mammary tumor development post-initiation, using immunocompromised mice.

**Results:**

Our findings suggest promotion of mammary tumor growth and metastasis by resveratrol at all concentrations tested in tumors derived from the low metastatic estrogen receptor (ER)α(-), ERβ(+) MDA-MB-231 and the highly metastatic ER(-) MDA-MB-435 cancer cell lines. Additionally, the activity of the migration/invasion regulator Rac, which we have previously shown to be regulated by resveratrol *in vitro*, was measured in tumors from resveratrol treated mice. Our results show a significant induction of tumoral Rac activity and a trend in increased expression of the Rac downstream effector PAK1 and other tumor promoting molecules following resveratrol treatment.

**Conclusion:**

Taken together, our findings implicate low concentrations of resveratrol in potential promotion of breast cancer. Therefore, this study illuminates the importance of further delineating resveratrol’s concentration dependent effects, particularly in breast cancer, before it can be tested in the clinic or used as a dietary supplement for breast cancer patients.

## Background

Dietary consumption of grapes has for long been associated with a decrease in the incidence of cardiovascular diseases and cancer. The beneficial properties of grapes and its products are attributed to polyphenolic compounds that have been shown to have cardioprotective, neuroprotective, anticancer, anti-inflammation, anti-ageing, and antimicrobial properties [1,2]. Among the grape and red wine polyphenols, resveratrol (3,4’ ,5-trihydroxy-*trans*-stilbene) has received a great deal of attention due to a plethora of *in vitro* and *in vivo* studies on its cancer chemopreventive and therapeutic properties. Resveratrol has been shown to affect initiation, promotion, and progression of tumorigenesis by modulating cell division and growth, apoptosis, angiogenesis, and metastasis [3-5].

Among the mediators of resveratrol’s anticancer effects, the most widely studied are the mitogen activated protein kinase (MAPK), phosphatidyl inositide 3-kinase (PI3-K)/Akt, and Nuclear Factor kappa B (NFkB) pathways [4,5]. Resveratrol is particularly important for breast cancer since it has been shown to exert both estrogenic or antiestrogenic effects and binds to estrogen receptors ERα and ERβ with comparable affinity, but with 7,000-fold lower affinity than estrogen [6-8]. Resveratrol-mediated regulation of extracellular regulated kinase (ERK)1/2, c-Jun N-terminal kinase (JNK), and p38 MAPKs has been shown to promote apoptosis of breast cancer cells [9-11]. Resveratrol inhibited cell proliferation and ERα mRNA and protein levels in MCF-7 breast cancer cells through a mechanism dependent on p38 MAPK and p53 regulation [12]. In addition to inhibition of cancer initiation and progression, resveratrol has also been implicated in reduction of metastasis. Resveratrol has been shown to inhibit cell migration/invasion and metastasis in several types of cancer, including breast cancer [13,14]. Moreover, resveratrol is able to chemosensitize several types of cancers to chemotherapy. Its potential as a chemosensitization agent is due to the regulation of many signaling molecules including drug transporters, cell survival and cell proliferation regulators, and members of the NFκB and signal transducer and activator of transcription (STAT)3 signaling pathways [15].

However, most studies on the anti-cancer activities of resveratrol have focused on high, non-physiologically relevant, concentrations; whereas the effects of low concentrations, achieved through the diet, on cancer progression have been poorly studied. Our group has shown that depending on the concentration used, resveratrol can act as an estrogenic or an anti-estrogenic compound. We have reported an inhibitory role for high concentrations of resveratrol and a promotional role for estrogen and low concentrations of resveratrol in breast cancer cell migration, extension of actin structures that promote cell migration, and the activity of Rac, a key signaling protein that promotes the actin cytoskeleton rearrangements driving cell migration [16,17].

Despite its many beneficial effects, resveratrol’s bioavailability is low as a result of its rapid metabolism in mammals [3]. However, due to its therapeutic potential, the use of novel delivery systems to increase resveratrol’s bioavailability is being explored [18,19]. In mouse models of cancer, resveratrol has shown variable results depending on the concentration, where early reports showed that resveratrol prevented mammary tumorigenesis and decreased mammary growth of MDA-MB-231 mammary tumors [6,20]. However, recent studies have shown either no effect of treatment with resveratrol *in vivo*[21,22] or pro-cancer effects in a MDA-MB-435 model, where resveratrol abrogated the effects of paclitaxel treatment [23].

A number of clinical trials are ongoing on the potential of resveratrol as an anti-cancer compound [24,25]. However, there is a pressing need to further understand the effects of resveratrol in pre-clinical studies, especially with established cancers, given that cancer patients often use resveratrol as a dietary supplement. Because of its low bioavailability, and since resveratrol’s dual estrogenic/antiestrogenic role seems to be concentration dependent, it is also important to delineate its anticancer effects at a broad range of concentrations.

Herein we have investigated the effects of resveratrol in the regulation of mammary tumor growth and metastasis at a range of concentrations in a mouse model of breast cancer, using the low metastatic ERα(-), ERβ(+) MDA-MB-231 and the highly metastatic ER(-) MDA-MB-435 cancer cell lines. The concentrations selected for this study range from dietary to pharmacological, where it is possible to achieve 0.5-5 mg/kg BW resveratrol concentrations via dietary consumption. In our study, in mice with an average bodyweight of 20 g, 5 mg/kg BW of resveratrol in a 100-μL gavage volume equates to ~4.38 μM resveratrol. These concentrations may be found in dietary components rich in grape polyphenols such as red wine [14]. Interestingly, our findings implicate low concentrations of resveratrol in promotion of mammary tumor growth and metastasis, and identify Rac and other cancer-promoting molecules as potential regulators of resveratrol-mediated effects in mammary tumors.

## Methods

### Animals

Hairless (severe combined immunodeficiency) SCID or athymic nu/nu (or nude) female mice, 5 to 6 wk old (Charles River Laboratories, Inc., Wilmington, MA) were maintained under pathogen-free conditions in Hepa-filtered cages under controlled light (12 h light and dark cycle), temperature (22-24°C), and humidity (25%). Throughout the experiment, the animals were provided with sterile AIN 76-A phytoestrogen-free diet (Tek Global, Harlan Teklad, Madison, WI) and water *ad libitum*. This project was approved by the Institutional Animal Care and Use Committee, Universidad Central del Caribe.

### Tumor establishment

Green fluorescent protein (GFP) tagged-MDA-MB-231 (ERα(-), ERβ(+)) or a metastatic variant of GFP-MDA-MB-435 (ER (-)) cells (~ 1 × 10^6^) in Matrigel (BD Biosciences, San Jose, CA) were injected into the fourth right mammary fat pad (only one site per mouse) under isofluorane inhalation to produce orthotopic primary tumors as described in [26]. After tumor establishment (1 wk post-inoculation), animals were randomly divided into experimental treatment groups (n=10-12 per treatment group). The GFP-MDA-MB-231 cells were inoculated into SCID mice, while the GFP-MDA-MB-435 cells were inoculated into nude mice. Both cell lines were a kind gift of Dr. Danny Welch, The University of Alabama at Birmingham, AL. SCID mice were used for the study conducted with MDA-MB-231 cells to ensure uniform tumor take. For the MDA-MB-435 study, nude mice were used as we previously obtained uniform tumor take with this model [26]. Moreover, the aggressiveness and high metastatic potential of the MDA-MB-435 cell line would have caused increased tumor burden in the SCID strain. All injected sites developed into palpable tumors and none regressed below the original signal.

The origin of MDA-MB-435 cell line has been questioned by studies that show expression of melanoma-associated genes [27]. However, MDA-MB-435 cells are derived from a breast cancer patient, express breast differentiation-specific proteins, and secrete milk lipids [28]. Therefore, the simplest conclusion is that MDA-MB-435 is a very poorly differentiated breast carcinoma. A recent publication supports this idea by providing evidence that suggests the MDA-MB-435 cell line is indeed a poorly differentiated, aggressive breast cancer cell line that expresses both epithelial and melanocytic markers [29].

### Diet administration

Hairless SCID or athymic nu/nu female mice were orally gavaged either with vehicle (90% neobee oil, 10% ethanol), or 0.5, 5, or 50 mg/kg body weight (BW) resveratrol in a 100 μL volume every day (5 days/wk). Treatments continued until sacrifice at day 108 for the study performed on GFP-MDA-MB-231 breast cancer cells and at day 44 for the study performed on GFP-MDA-MB-435 breast cancer cells. The study performed with GFP-MDA-MB-435 cells had to be terminated earlier due to high tumor burden caused by resveratrol in this highly metastatic and aggressive breast cancer cell line.

### Whole body fluorescence image analysis

Mammary tumor growth was quantified as changes in integrated density of GFP fluorescence, using methods developed by Hoffman and co-workers [30-32] and previously described by us in [26,33-35]. Mice (under anesthesia) were imaged one week following breast cancer cell inoculation (on the first day of diet administration) and once a week thereafter. A 300 Watt power source with two optical delivery systems fitted with excitation filters (470/40 nm) was used for whole body imaging of GFP fluorescence (LT99D2, Lightools Research, Encinitas, CA). Images were captured with a Spot II charge-coupled device (CCD) camera (Diagnostic Instruments, Sterling Heights, MI) mounted with a 530/25 nm emission filter (Chroma Technology, Rockingham, VT).

Tumor fluorescence intensities were analyzed using Image J software (National Institutes of Health, Bethesda, MD). The integrated density of each tumor was computed using threshold values set to detect the fluorescence from the primary tumor area and not the metastatic lesions. Relative tumor growth was calculated as the integrated density of fluorescence of each tumor on each imaging day relative to the integrated density of fluorescence of the same tumor on the first day of diet administration.

### Analysis of metastases

Following sacrifice, lungs, kidneys, livers, and bones (femurs) were excised and immediately stored in liquid N_2_. Stored organs were thawed and analyzed using an Olympus MV10 fluorescence macro zoom system microscope and images acquired with an Olympus DP71 digital camera as described in [26,34,35]. Each organ was imaged on both sides. The fluorescent lesions (green component of RGB images) were quantified for integrated density of fluorescent pixels using Image J software.

### Western blot analysis of tumors

Flash frozen MDA-MB-231-derived primary tumors were lysed using a homogenizer (Brinkmann Polytron, Mississauga, ONT, Canada) as previously reported by us [35]; and total protein quantified using the Precision Red protein assay kit (Cytoskeleton, Inc. Denver, CO). Equal total protein amounts were resolved on SDS-PAGE gels and western blotted using anti-Akt, anti-JNK, anti-PAK1, or anti-GFP (Cell Signaling Technology, Inc., Danvers, MA) antibodies. The integrated density of positive bands was quantified using Image J software and normalized with GFP to ensure quantification of tumor proteins.

### Western blot analysis of cells

Quiescent MDA-MB-231 or MDA-MB-435 cells were treated with vehicle, or 0.1, 0.5, or 5 μM resveratrol for 15 min. Cells were immediately lysed and total protein was quantified using the Precision Red protein assay kit (Cytoskeleton, Inc., Denver, CO). Equal total protein amounts were western blotted using anti-Akt, anti-phospho Akt^Thr308^, anti-JNK, anti-phospho JNK^Thr183/Tyr185^, anti-PAK1, or anti-phospho PAK1^Thr431/Thr402^ (Cell Signaling Technology, Inc., Danvers, MA) antibodies. The integrated density of positive bands was quantified using Image J software.

### Rac activity assays

Flash frozen mammary tumors were lysed using a homogenizer (Brinkmann Polytron, Mississauga, ONT, Canada) as previously described by us [35] and total protein was quantified using the Precision Red protein assay kit (Cytoskeleton, Inc. Denver, CO). Active Rac was pulled down using beads coupled to GST–p21-activated kinase (PAK)-Cdc42/Rac interactive binding (CRIB) motif (GST-PAK-PBD beads) (Cytoskeleton, Denver, CO) as described in [36], and detected from western blots immunostained with an anti-Rac antibody (Cell Signaling Technology, Inc., Danvers, MA). Positive bands were imaged using a VersaDoc system (Bio-Rad, Hercules, CA) and quantified using Image J software. Rac activity was determined as the Rac-GTP bound to the PAK–CRIB domain as a function of total Rac in a cell lysate.

### Statistical analysis

Data are expressed as the mean ± SEM. Statistical analyses were done using Microsoft Excel and GraphPad Prism®. Differences between means were determined using Student’s t-Test or one-way ANOVA with Dunnett's Multiple Comparison Test; and considered to be statistically significant at *p*≤0.05.

## Results and discussion

Our group has previously reported the dual effects of resveratrol in ER(-) breast cancer cell migration and invasion, while others have shown that resveratrol functions as an anti-estrogen in ER(+) mammary cancer cell lines and rodent models [6]. Our studies with ERα(-), ERβ(+) MDA-MB-231 breast cancer cell line demonstrated that resveratrol at 50 μM acts in an anti-estrogenic manner to reduce cell migration while, resveratrol at 5 μM acts similar to estrogen inducing cell migration, invasion, and formation of lamellipodia [36]. Lamellipodia are actin structures found at the leading edge of migrating cells that are under Rac regulation [37]. We reported that 50 μM resveratrol decreases Rac activity, whereas estrogen and 5 μM resveratrol increases Rac activity in breast cancer cells. Moreover, using breast cancer cells expressing dominant-negative Rac, we demonstrated that signaling to the actin cytoskeleton by low concentrations of resveratrol was under Rac regulation [36]. Since, breast cancers often lose ER expression during progression, we tested the effects of resveratrol in both ERβ(+) and ER(-) metastatic breast cancer cells MDA-MB-231 and MDA-MB-435 *in vivo*.

Many in vivo studies supporting the anti-cancer effects of resveratrol have used high, non-physiologically rele-vant, concentrations of resveratrol of 50-800 mg/kg BW [6,38-40]. However, the effects of low concentrations of resveratrol, which are critical for a better understanding of the possible effect of dietary resveratrol in cancer and for dosing recommendations, have been poorly addressed at the in vivo scenario. Therefore low, physiologically relevant, concentrations of resveratrol (0.5 and 5 mg/kg BW) and a moderate concentration (50 mg/kg BW) were used to test resveratrol’s effect on the progression of breast cancer. 

The bioavailability of resveratrol is considered to be low as per data on plasma levels of resveratrol following dietary consumption. Studies have shown that administration of 20 mg/kg resveratrol to rats, mice, or rabbits demonstrated a very short half-life (10 minutes) and reached circulating levels of less than 1 μM very rapidly [20]. Mice administered 100 mg/kg of resveratrol achieved a maximum serum concentration of 2277 ng/mL at 0.25 h and ~42 ng/mL at 24 h [18]. However, others have shown that, administration of as little as 50 μg/kg body weight of resveratrol per day for 3 months to rats achieved serum levels as high as 8.0 μM [39]. In the present study, we administered low to moderate (0.5, 5, and 50 mg/kg BW) concentrations of resveratrol daily 5× a week for 1.5 and 4 months in the MDA-MB-435 and MDA-MB231 studies, respectively. Therefore, we expect the resveratrol levels in the plasma to be low but consistent, and higher in the mammary tissue, as per previous studies that have shown resveratrol levels in plasma are low due to high absorption by tissues [41].

**Figure 1:**
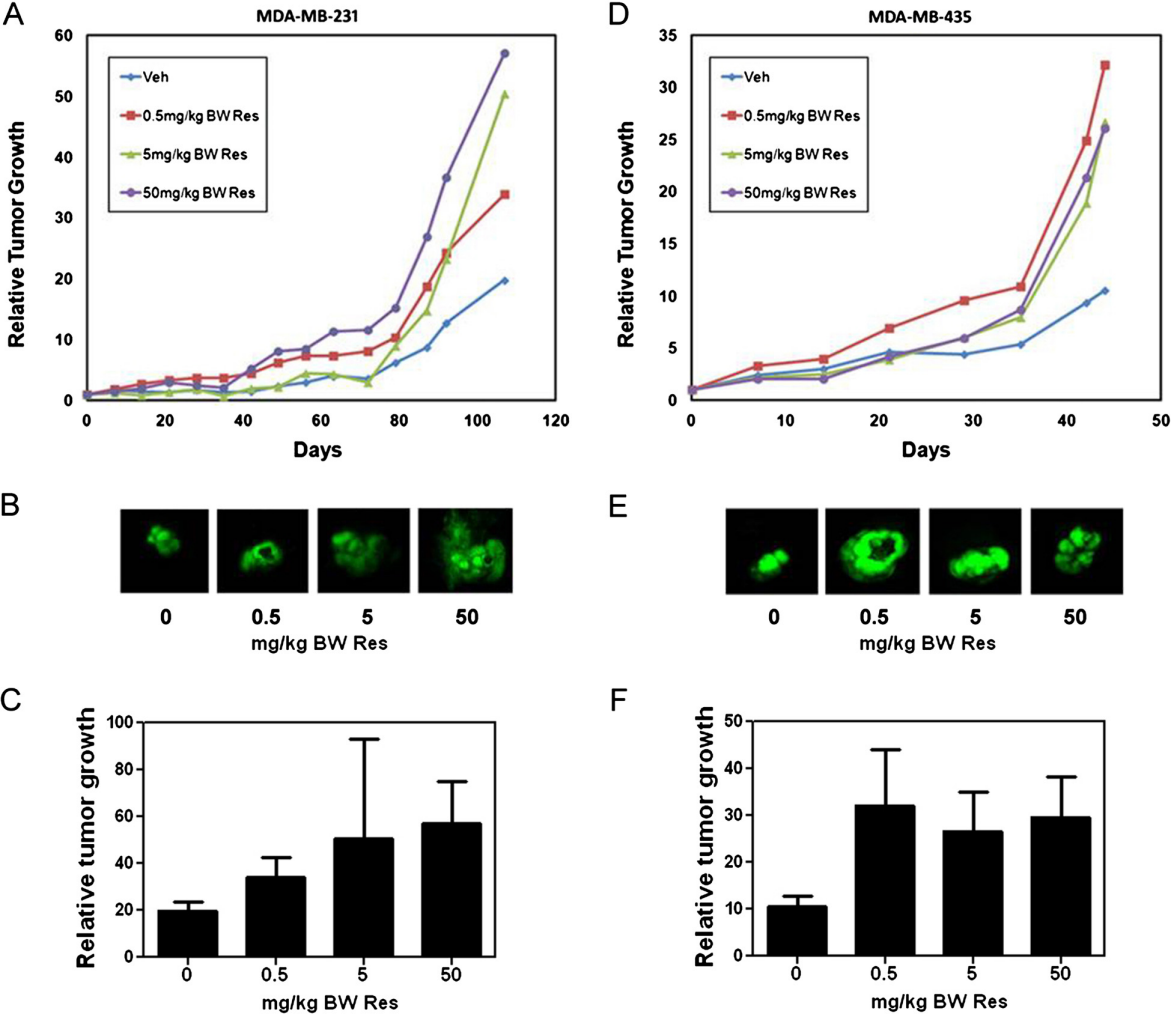
**Effect of resveratrol on the growth of mammary fat pad tumors.** 1 × 10^6^ GFP-tagged MDA-MB-231 or MDA-MB-435 cells in Matrigel:DMEM (1:1) were inoculated at the mammary fat pad of female SCID (MDA-MB-231) or athymic nude mice (MDA-MB-435). One week following injection, mice were fed vehicle (Veh) or 0.5, 5, or 50 mg/kg BW resveratrol (Res) 5 times a week by oral gavage. Whole body fluorescence images were acquired once a week. Relative tumor growth was calculated as the integrated density of fluorescence on each day of imaging as a function of the integrated density of fluorescence of the same tumor on day 1 of treatment administration. (**A**), Average relative GFP-MDA-MB-231 tumor growth as a function of days. (**B**), Representative images of GFP-MDA-MB-231 tumors following vehicle or Res diets at day 108. (**C**), Relative GFP-MDA-MB-231 tumor growth for each treatment group at day 108 ±SEM. (**D**), Average relative GFP-MDA-MB-435 tumor growth as a function of days. (**E**), Representative images of GFP-MDA-MB-435 tumors following vehicle or Res diets at day 44. (**F**), Relative GFP-MDA-MB-435 tumor growth for each treatment group at day 44 ±SEM. Differences between means were determined using Student’s t-Test or one-way ANOVA with Dunnett's Multiple Comparison Test.

In SCID mice with MDA-MB-231 mammary fat pad tumors, oral gavage of resveratrol resulted in a nonsignificant, but evident increase in tumor growth of ~2-fold at 0.5 mg/kg BW resveratrol and ~2.5-fold at 5 and 50 mg/kg BW resveratrol (Figure 1A, B, and C). Similarly, the growth of mammary fat pad tumors established in nude mice from the more aggressive and highly metastatic breast cancer cell line MDA-MB-435 was increased by resveratrol at all the concentrations tested. In the MDA-MB-435 study, the concentration that showed the greater effect was 0.5 mg/kg BW resveratrol with a ~3-fold increase, whereas 5 and 50 mg/kg BW resveratrol increased tumor growth by ~2.5-fold (Figure 1D, E, and F). This increase in tumor growth presented with a *p*>0.05 compared to controls*.* This high probability may be due to the heterogeneity of individual tumor growth, because when we re-group into growing and poorly growing groups as cited in [42]*,* there was a significant difference of 0.02 in the 5 mg/kg BW resveratrol group compared to vehicle controls. Moreover, administration of resveratrol at all concentrations tested did not result in significant differences in mouse weight among the resveratrol treated groups compared to vehicle controls (Additional file 1: Figure S1).

Even though our previous study, where we administered 5 mg/kg BW resveratrol in combination with equal amounts of quercetin and catechin, demonstrated reduced tumor growth and metastasis [26,34], in this study we found resveratrol alone induced a marked trend of increase in tumor growth, especially by the lower concentrations; thus, indicating a deleterious effect of resveratrol on breast cancer progression. This may suggest an alternate mechanism where resveratrol alone acts to promote tumor growth.

Our results highlight the importance of attaining a better understanding of the effects of resveratrol at low concentrations. Also noteworthy is the fact that 50 mg/kg BW resveratrol, which in other studies has been shown to reduce tumor growth in ovarian cancer and hepatocarcinoma [38,40], induced tumor growth in both (MDA-MB-231 and MDA-MB-435) breast cancer cells. This suggests that resveratrol effects are not only concentration dependent but can also vary depending on the cell and cancer type.

**Figure 2:**
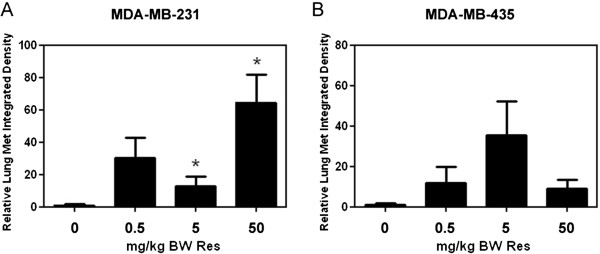
**Effect of resveratrol on lung metastasis.** Following necropsy, lungs were excised from mice with GFP-MDA-MB-231 or GFP-MDA-MB-435 mammary tumors that received vehicle or 0.5, 5, or 50 mg/kg BW Res diets and analyzed for metastases by fluorescent microscopy followed by quantitative image analysis. (**A**) and (**B**), Relative integrated density (±SEM) of fluorescent metastatic foci for vehicle or Res treated mice from the study performed with (**A**), MDA-MB-231 and (**B**), MDA-MB-435 cells (2-9 mice/group). Differences between means were determined using Student’s t-Test. Asterisk denotes statistical significance at *p≤*0.05.

To investigate the role of resveratrol on breast cancer metastasis, lungs, livers, kidneys, and bones from mice bearing MDA-MB-231 and MDA-MB-435 mammary tumors were harvested at necropsy for identification of fluorescent metastatic foci. Mice from the MDA-MB-231 study only presented with lung metastasis; whereas, mice from the MDA-MB-435 study presented with metastasis to all harvested organs due to the highly metastatic potential of this cell line. Resveratrol dramatically induced lung metastasis in both studies. In mice bearing MDA-MB-231 derived mammary tumors, resveratrol at 0.5, mg/kg BW caused a 30-fold increase, and at 5 and 50 mg/kg BW, a significant 13- and 65-fold increase in lung metastasis after 108 days (Figure 2A). Likewise, in mice bearing MDA-MB-435 tumors, resveratrol at 0.5, 5, and 50 mg/kg BW caused an 11-, 36-, and 9-fold increase in lung metastasis, respectively, after only 44 days (Figure 2B). In the case of kidneys and bone, mice from the vehicle group did not present with metastasis while resveratrol-treated mice developed metastases (Table 1). Metastasis to the kidneys was observed in 14%, 10%, and 22% of mice in 0.5, 5, and 50 mg/kg BW resveratrol treatment groups, respectively; whereas, metastasis to the bone was observed in 20% and 22% of mice treated with 5 and 50 mg/kg BW resveratrol, respectively. Finally, liver metastases were identified in a similar percentage of mice from each treatment group, including vehicle control. However, the average integrated density was 10- and 2.9- fold higher in 0.5 and 5 mg/kg BW resveratrol treated mice (Table 1), suggesting a higher number and/or larger metastatic foci in mice treated with low concentrations of resveratrol when compared to vehicle treated mice. Overall, the percentage of mice presented with metastasis was low for statistical comparisons due to the short duration of the study which, because of high tumor burden, had to be terminated after only 44 days. Regardless, our findings strongly suggest that resveratrol at low concentrations can promote mammary tumor growth and metastasis.

**Table 1:** Effects of resveratrol on metastasis of GFP-MDA-MB-435 mammary tumors

	**Percentage of mice with metastases**				**Average integrated density of fluorescent metastatic foci**			
	**Vehicle**	**Resveratrol (mg/kg BW)**			**Vehicle**	**Resveratrol (mg/kg BW)**		
		**0.5**	**5**	**50**		**0.5**	**5**	**50**
**Kidneys**	0% (0/7)	14% (1/7)	10% (1/10)	22% (2/9)	0	0.21	0.12	0.29
**Liver**	29% (2/7)	29% (2/7)	10% (1/10)	22% (2/9)	0.24	2.4	0.70	0.20
**Bone**	0% (0/7)	0% (0/7)	20% (2/10)	22% (2/9)	0	0	0.038	0.02

**Figure 3:**
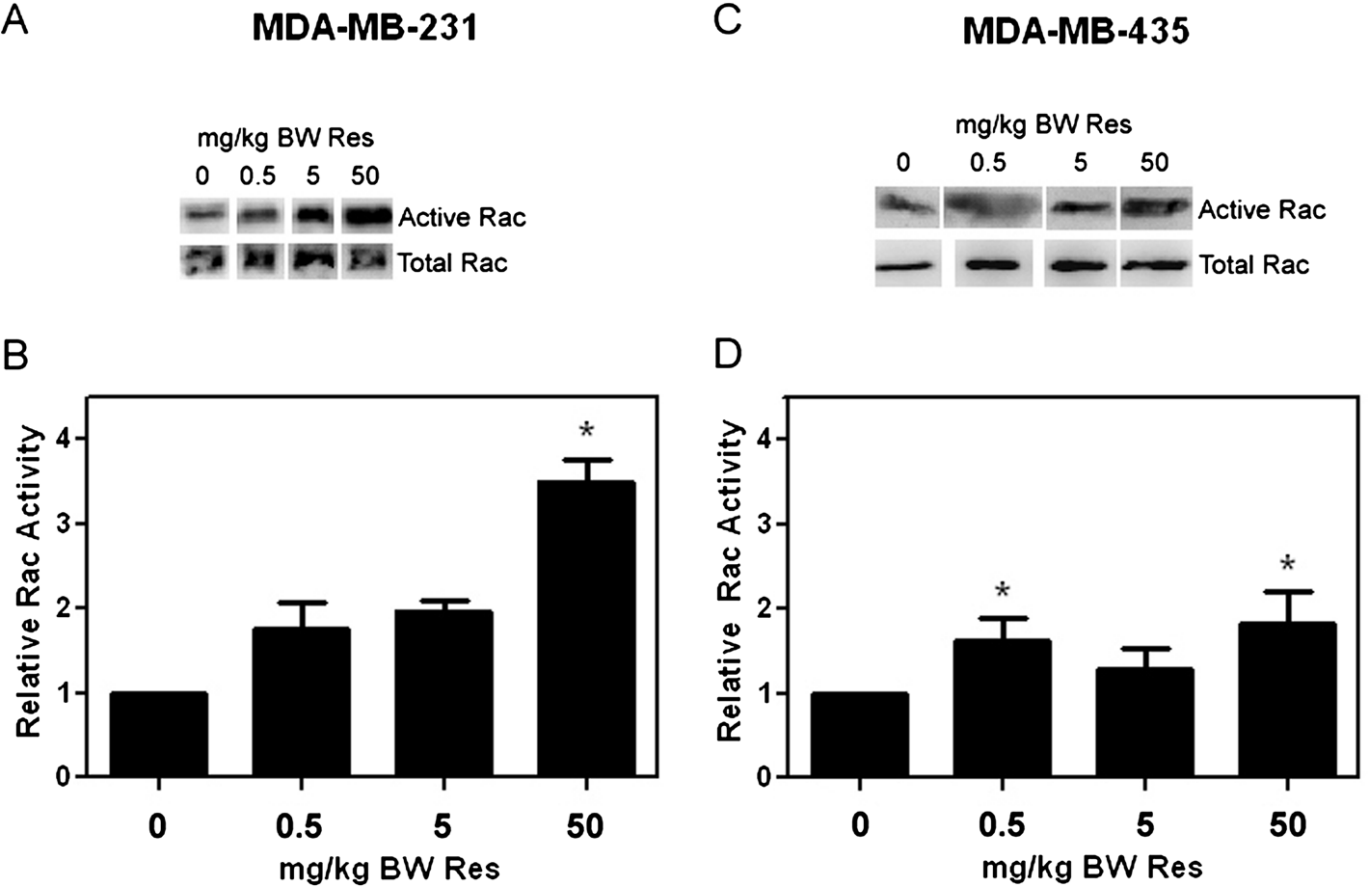
**Rac activity in resveratrol treated tumors.** GFP-MDA-MB-231 or GFP-MDA-MB-435 mammary tumors from mice that received vehicle or 0.5, 5, or 50 mg/kg BW Res diets were lysed. PAK–PBD–GST beads were used to pull down active (GTP-bound) Rac from tumor lysates. Active and total Rac levels were detected by western blot analysis with anti-Rac antibody. Rac activity (active Rac/total Rac) relative to vehicle control as quantified from densitometric scans of western blot analyses is presented for the study performed with (**A** and **B**), MDA-MB-231 and (**C**-**D**), MDA-MB-435 cells. **(A)** and **(C)**, Representative western blots for active (GTP-bound) Rac in the upper row and total tumoral Rac in the lower row for the MDA-MB-231 and the MDA-MB-435 studies, respectively. **(B)** and **(D)**, Average relative Rac activity for the MDA-MB-231 and the MDA-MB-435 studies, respectively. Error bars represent SEMs from 2-4 tumors per group. Differences between means were determined using Student’s t-Test. Asterisk denotes statistical significance at *p≤*0.05.

Since resveratrol-mediated regulation of breast cancer cell migration/invasion was previously shown by us to be dependent on signaling from the small GTPase Rac [36], we investigated Rac activation status in MDA-MB-231 and MDA-MB-435-derived mammary tumors. In MDA-MB-231 mammary tumors, resveratrol at 50 mg/kg BW significantly increased Rac activity by 3.5-fold; whereas 0.5 and 5 mg/kg BW caused a non-significant increase in Rac activity of 1.75 and 2-fold, respectively (Figure 3A). On the other hand, in MDA-MB-435-derived tumors, resveratrol at 0.5 and 50 mg/kg BW significantly increased Rac activity by 1.6- and 1.8-fold, respectively (Figure 3B). Our results suggest that resveratrol-mediated induction of metastasis might be, at least partly due to increased Rac activity.

**Figure 4:**
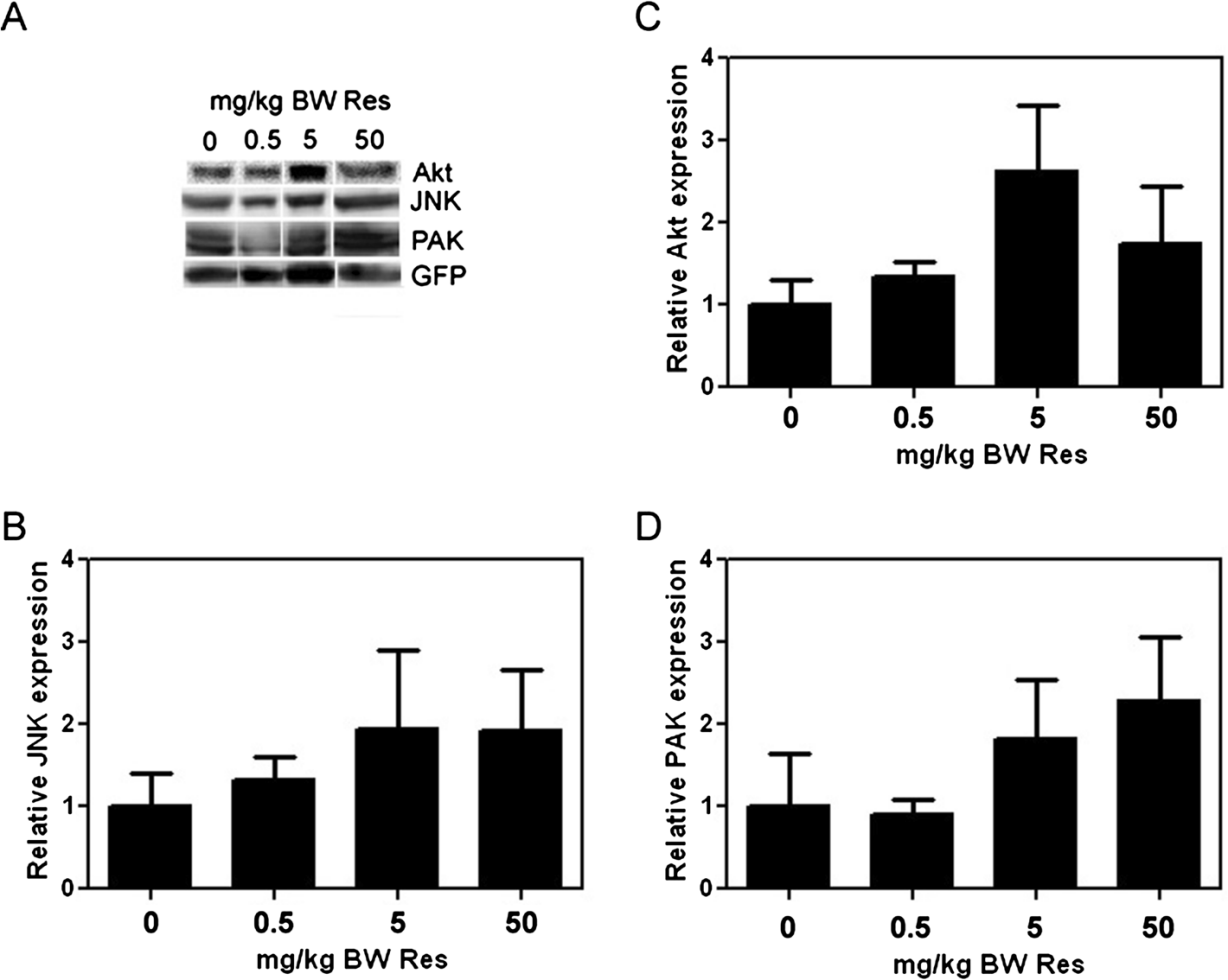
**Effect of resveratrol on Akt, JNK, and PAK expression in mammary tumors.** Total lysates of MDA-MB-231-derived mammary tumors from vehicle- and Res-treated mice were western blotted with anti-Akt, anti-JNK, or anti-PAK1 antibodies. Fold changes in protein expression were calculated from integrated density of positive bands and are presented relative to vehicle. Equal total protein content as well as confirmation of human cancer cells in the mouse tumor extracts was maintained by expressing the integrated density of each band as a function of the integrated density of the GFP band from the same tumor extract. **(A)**, Representative western blots. **(B)**, **(C)**, and **(D)**, Relative Akt, JNK, and PAK1 expression, respectively. Error bars represent SEMs from 2-4 tumors per group. Differences between means were determined using Student’s t-Test.

We also investigated the effects of resveratrol in the tumoral expression of Akt and the MAPK jun kinase (JNK), key proteins in survival signaling and cell proliferation that are commonly disregulated in human cancers and have been shown to be regulated by resveratrol [4,6;10-12;38-40]. In addition, we studied the expression of the Rac downstream effector p-21 activated kinase 1 (PAK1), implicated in the regulation of cell motility/migration and therefore, metastasis. PAKs are also relevant for the control of other cellular processes important for tumorigenesis such as survival, proliferation and gene transcription [41], via regulation of Akt and MAPKs pathways [42]. We analyzed Akt, JNK, and PAK1 protein expression in MDA-MB-231 derived mammary tumors by western blot. Our results show a trend in upregulation (1.75-2.5-fold) of these kinases in tumors from mice that received resveratrol at 5 and 50 mg/kg BW; thus, implicating the PI3-K/Akt, MAPK, and Rac/PAK pathways in potential effects of resveratrol on mammary tumor growth and metastasis (Figure 4).

**Figure 5:**
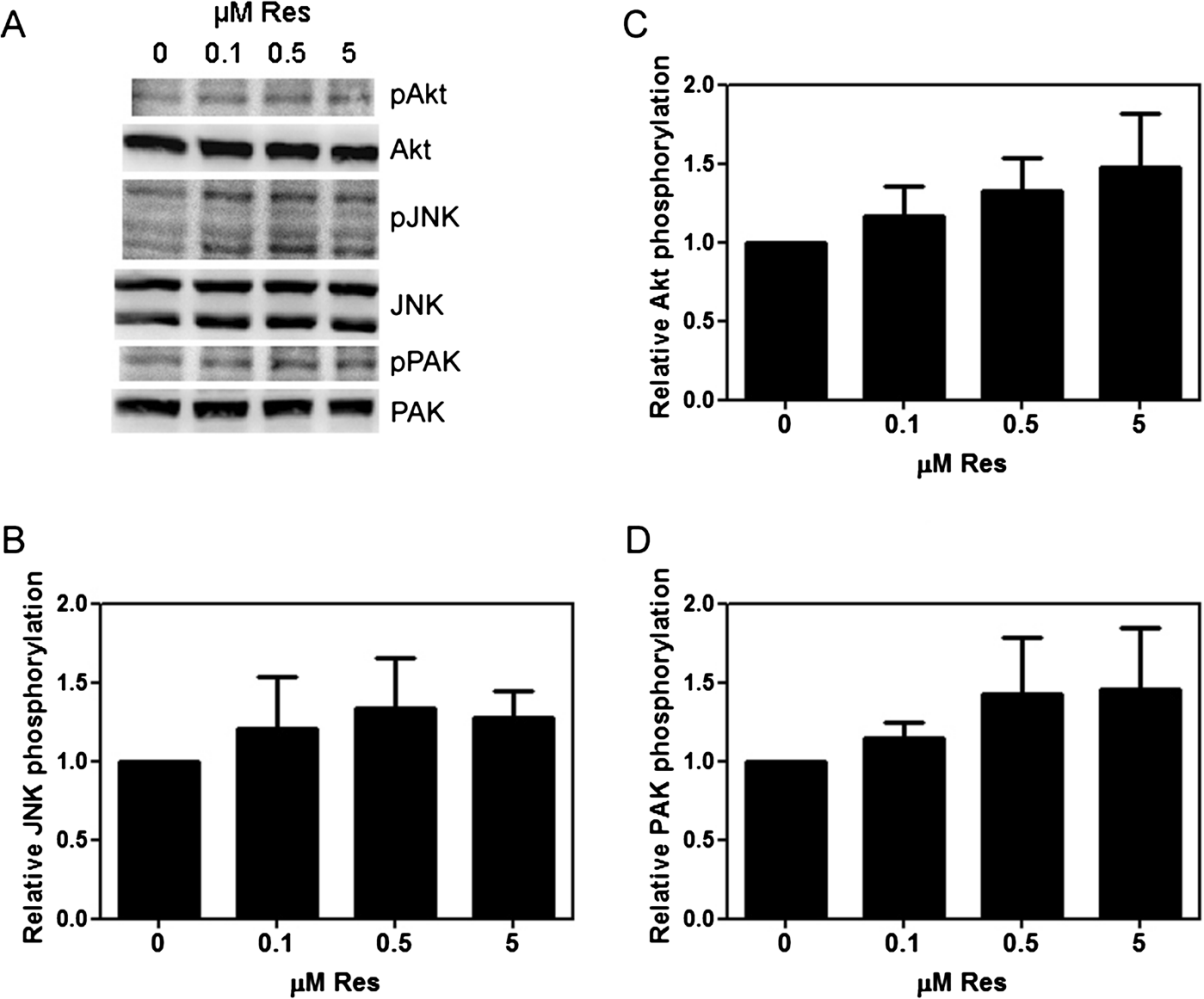
**Effect of resveratrol on Akt, JNK, and PAK1 activity in MDA-MB-231 breast cancer cells.** Confluent MDA-MB-231 cells were serum starved for 24 h, treated with Veh, 0.1, 0.5, or 5 μM resveratrol (Res) for 15 min, lysed immediately, and western blotted for active and total proteins: phospho-Akt^Thr308^ and Akt, phospho-JNK^Thr183/Tyr185^ and JNK, or phospho-PAK1^Thr431/Thr402^ and PAK1. Fold changes in protein activity were calculated from integrated density of positive bands and are presented relative to vehicle. **(A)**, Representative western blots. **(B)**, **(C)**, and **(D)**, Relative Akt, JNK, and PAK1 activity (phosphorylated protein/total protein), respectively (n=2-3). Error bars represent SEMs. Differences between means were determined using Student’s t-Test.

To test whether the increased expression of Akt, JNK, and PAK1 resulted in parallel increases in their activity, we tested the effect of low concentrations of resveratrol on the active phosphorylated levels of these signaling proteins. Quiescent MDA-MB-231 or MDA-MB-435 cells were treated with 0.1, 0.5, and 5 μM resveratrol for 15 min and the lysates western blotted using total or phsopho-specific antibodies to Akt, JNK, and PAK1. In parallel with increased expression of total proteins from the MDA-MB-231 mammary tumors in mice treated with resveratrol (Figure 4), we observed increased phosphorylation of Akt, JNK, and PAK1 by resveratrol at all concentrations tested in MDA-MB-231 cells (Figure 5), but not in MDA-MB-435 cells (data not shown). Therefore, the upregulated expression and activities of the Rac effectors PAK, JNK, and Akt, are expected to contribute to the observed increase in tumor growth and metastasis in response to resveratrol.

Even though resveratrol at high concentrations inhibits Akt and MAPK activities, at low concentrations, resveratrol has been previously shown to promote proliferation in human cancer cells and to induce Akt and MAPKs, among other tumorigenic signaling proteins [43]. Resveratrol modulates a plethora of signaling pathways important for tumor progression; and therefore, it would be too simplistic to limit resveratrol’s cancer promoting (or inhibiting) properties to a few molecules or a single signaling pathway [44,45]. Thus, it is more reasonable to think that the collective activity of resveratrol, rather than a single effect, is responsible for our results. In addition to modulation of steroid receptor signaling and crosstalk with growth factor receptors that can affect both signaling and transcriptional activities [46], resveratrol was recently shown to exhibit dose dependent regulation of Wnt signaling and the expression of Wnt target genes [47]. Since resveratrol affected mammary tumor growth in both ERα(-), ERβ(+) MDA-MB-231 and ER(-) MDA-MB-435 cancer cells, ER independent mechanisms may be responsible for our intriguing findings. Such mechanisms can include: 1) the regulation of ER related receptors that are active in ER(-) breast cancers [48] and are regulated by phytoestrogens [49], or 2) the collective regulation of multiple signaling and transcriptional pathways.

## Conclusions

Resveratrol is widely known for its anti-cancer properties. However, our study implicates low concentrations of resveratrol on promotion of breast cancer progression and metastasis and implicates tumorigenic and metastatic signaling pathways in resveratrol’s tumor promoting effects. The present study clearly demonstrates that resveratrol, depending on the dose, can induce growth and metastasis of breast cancers. Therefore, prior to dietary recommendations of resveratrol or clinical trials as an anti-cancer therapeutic, it is critical to have a better understanding of resveratrol effects at physiological concentrations. Moreover, our findings serve to caution patients or physicians in the use of this grape polyphenol as an alternative and/or complementary medicine for breast cancer treatment.

## Competing interests

The authors declare that they have no competing interests.

## Authors’ contributions

LCP contributed to the experimental design, conducted all experiments and analyzed and interpreted data. LCP also wrote the manuscript. LAC was involved with critical revision of the manuscript. SD contributed to the experimental design, directed the research, assisted with animal protocols, and data analysis and interpretation. She also contributed to writing and critical revision of the manuscript. All authors read and approved the final manuscript.

## Pre-publication history

The pre-publication history for this paper can be accessed here:

http://www.biomedcentral.com/1472-6882/13/6/prepub

## Supplementary Material

Additional file 1 **Figure S1.** Effect of resveratrol on mice weight. GFP-tagged MDA-MB-231 or MDA-MB-435 cells (1 × 10^6^) in Matrigel:DMEM (1:1) were inoculated at the mammary fat pad of female SCID (MDA-MB-231) or athymic nude mice (MDA-MB-435). One week following injection, mice were fed vehicle (Veh) or 0.5, 5, or 50 mg/kg BW resveratrol (Res) 5 times a week by oral gavage. Mice weights were recorded every two weeks to monitor toxicity and are presented for (**A**) the MDA-MB-231 study and (**B**) the MDA-MB-435 study, as a function of days. Differences between means were determined using Student’s t-Test. Click here for file
