# Supplementary figures and images for: Dietary grape polyphenol resveratrol increases mammary tumor growth and metastasis in immunocompromised mice
Source: BMC Complement Altern Med. 2013 Jan 8;13:6. doi: 10.1186/1472-6882-13-6 (PMC3544562; doi:10.1186/1472-6882-13-6)

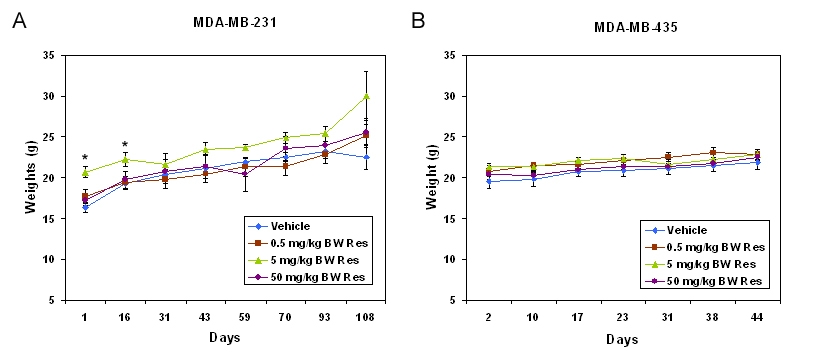

Supplement: Additional file 1 — Figure S1. Effect of resveratrol on mice weight. GFP-tagged MDA-MB-231 or MDA-MB-435 cells (1 × 10^6^) in Matrigel:DMEM (1:1) were inoculated at the mammary fat pad of female SCID (MDA-MB-231) or athymic nude mice (MDA-MB-435). One week following injection, mice were fed vehicle (Veh) or 0.5, 5, or 50 mg/kg BW resveratrol (Res) 5 times a week by oral gavage. Mice weights were recorded every two weeks to monitor toxicity and are presented for (A) the MDA-MB-231 study and (B) the MDA-MB-435 study, as a function of days. Differences between means were determined using Student’s t-Test. [file 1472-6882-13-6-S1.jpeg]
